# Impact of Multiple Factors on the Degree of Tinnitus Distress

**DOI:** 10.3389/fnhum.2016.00341

**Published:** 2016-06-29

**Authors:** Petra Brüggemann, Agnieszka J. Szczepek, Matthias Rose, Laurence McKenna, Heidi Olze, Birgit Mazurek

**Affiliations:** ^1^Tinnitus Center, Universitätsmedizin BerlinBerlin, Germany; ^2^Department of Otorhinolaryngology, Universitätsmedizin BerlinBerlin, Germany; ^3^Department of Internal Medicine and Psychosomatics, Universitätsmedizin BerlinBerlin, Germany; ^4^Royal National Throat Nose and Ear Hospital, University College HospitalsLondon, UK

**Keywords:** the grade of tinnitus-related distress, the psychological distress, physical or psychological discomfort, social parameters, multi-level analyses

## Abstract

**Objective:** The primary cause of subjective tinnitus is a dysfunction of the auditory system; however, the degree of distress tinnitus causes depends largely on the psychological status of the patient. Our goal was to attempt to associate the grade of tinnitus-related distress with the psychological distress, physical, or psychological discomfort patients experienced, as well as potentially relevant social parameters, through a simultaneous analysis of these factors.

**Methods:** We determined the level of tinnitus-related distress in 531 tinnitus patients using the German version of the tinnitus questionnaire (TQ). In addition, we used the Perceived Stress Questionnaire (PSQ); General Depression Scale Allgemeine Depression Skala (ADS), Berlin Mood Questionnaire (BSF); somatic symptoms inventory (BI), and SF-8 health survey as well as general information collected through a medical history.

**Results:** The TQ score significantly correlated with a score obtained using PSQ, ADS, BSF, BI, and SF-8 alongside psychosocial factors such as age, gender, and marital status. The level of hearing loss and the auditory properties of the specific tinnitus combined with perceived stress and the degree of depressive mood and somatic discomfort of a patient were identified as medium-strong predictors of chronic tinnitus. Social factors such as gender, age, or marital status also had an impact on the degree of tinnitus distress. The results that were obtained were implemented in a specific cortical distress network model.

**Conclusions:** Using a large representative sample of patients with chronic tinnitus permitted a simultaneous statistical measurement of psychometric and audiological parameters in predicting tinnitus distress. We demonstrate that single factors can be distinguished in a manner that explains their causative association and influence on the induction of tinnitus-related distress.

## Introduction

### Multi-dimensional causes of tinnitus

Tinnitus distress is a multidimensional phenomenon that can be associated with problems such as difficulties with concentration, insomnia, or negative thinking which can amplify it in vicious cycles (Zirke et al., 2013; McKenna et al., 2014). Complex relationships between tinnitus, other stressors, and social factors such as education, age, and gender have been reported in the literature (Nondahl et al., 2012; Seydel et al., 2013). Furthermore, tinnitus distress seems to be more commonly reported by patients from lower social classes. Tinnitus percept results from an imbalance between excitatory and inhibitory functions in the auditory pathway (Eggermont and Roberts, 2012). Cortical mental hyperactivity can exacerbate tinnitus distress and turn it into a chronic condition. Sub-cortical structures that participate in the emotional processing of auditory signals may contribute to negative interpretations of the noise and can amplify it in response to stress, anxiety, or other factors (Leaver et al., 2011). Tinnitus can also be worsened by various orthopedic conditions (Biesinger et al., 2008).

### Influence of psychological and somatic complaints

A patient's level of awareness of tinnitus depends on multiple sound-processing mechanisms (Hesse, 2008) and is considered a result of selective attention combined with an absence of habituation (Hallam et al., 2004). From a psychological point of view, reducing negative interpretations of the noise and changing certain types of behavior will generally reduce emotional distress while increasing habituation, to render the tinnitus less intrusive (Wallhausser-Franke et al., 2012; McKenna et al., 2014). The neurophysiological model of tinnitus assumes that it originates in the inner ear, followed by detection to the subcortex and further steps of processing, perception, and evaluation to various cortical areas (Jastreboff, 1999). A current model views specific thalamic nuclei as filters or gates where decisions about the conscious processing of tinnitus are made (Rauschecker et al., 2010). While these psychological and neurophysiological models appear similar, they emphasize different conscious and unconscious processes in attempting to explain the experience of tinnitus distress and the treatments they propose, reflecting differences in their philosophical underpinnings (Hallam et al., 2004).

Whatever the mechanism that generates tinnitus distress, it is widely thought to involve a link between overly negative thoughts and emotions and auditory stimuli. Patients recite a common litany of despair, persecution, hopelessness, a loss of enjoyment in life, a desire for peace and quiet, and often beliefs that others do not understand (Wilson and Henry, 1998). Tinnitus distress often leads to negative thinking about the disease itself and its role in a patient's life (Henry and Wilson, 1995; Andersson and Westin, 2008). In advanced stages, tinnitus distress amplifies negative thoughts about the disease itself, starting a cycle that can lead to an even greater emotional decline. At some point 65% of tinnitus patients are diagnosed with depression, anxiety, or another mental condition (Hiller and Goebel, 1992; Andersson and Kaldo, 2004; Zirke et al., 2013).

Recently these links were highlighted in a report on the association between catastrophic thinking, high subjective judgments of the loudness of the tinnitus, poorer coping, the appearance of symptoms of depression, and more frequent medical visits (Weise et al., 2013). Catastrophic interpretations of tinnitus were also associated with fear (Cima et al., 2011; Pattyn et al., 2016). Several other studies have suggested a link between a poor emotional state and increased tinnitus distress (Halford and Anderson, 1991; Zoger et al., 2006; Wallhausser-Franke et al., 2012; Milerova et al., 2013). The overall process appears to include an increase in stress arousal, culminating in selective attention, and more monitoring of tinnitus, which in turn increases its detection and creates a vicious cycle (McKenna et al., 2014). This is consistent with observations that catastrophic interpretations lead to fear, increased attention devoted to the tinnitus, and a decrease in quality of life (Cima et al., 2011). Another result is that patients change their behavior in ways that are intended to reduce the threat, but in fact perpetuate it or increase negative thinking even more (Hesser and Andersson, 2009; Kleinstauber et al., 2013).

Such observations have strengthened the notion that links between limbic and auditory areas of the brain play a significant role in processing tinnitus and generating accompanying distress. The perception of tinnitus must follow from some neurophysiological process that sensitizes cognitive areas (Zenner, 2003). This model suggests that neuronal plasticity lowers normal perceptual thresholds and makes tinnitus audible, and the neural network responds by making subject, emotional, and somatic/motoric functions hyper-reactive. In the diathesis-stress model and similar frameworks, tinnitus is regarded as a stressor and may be aggravated by the elevated levels of stress associated with a vulnerable constitution (Andersson and McKenna, 1998; Kroner-Herwig et al., 2006).

### Comorbidity

Many polygenic diseases with high rates of comorbidity are considered to incorporate a significant stress component (Chrousos, 2009). The influence of stress on the auditory system has been detected on molecular and cellular levels (Ma et al., 1995; Horner, 2003; Mazurek et al., 2010a, 2012; Kraus and Canlon, 2012). Stress can arise from a number of sources, including an individual's general disposition or psychological coping mechanisms. The severity of tinnitus has been related to the personality traits of perfectionism (Andersson et al., 2005) and anxiety sensitivity (Andersson and Kaldo, 2004; Hesser and Andersson, 2009). Optimism, as might be expected, is negatively associated with tinnitus distress (Vollmann et al., 2013).

Establishing a causal relationship between such personality variables and tinnitus distress is challenging. The appearance of traits of anxiety shortly after the onset of tinnitus has been used as a predictor that the distress will be greater 6 months later (Langenbach et al., 2005) suggesting that people who are already emotionally strained are more likely to be distressed by tinnitus. An additional relationship has been found between distress, quality of life, and Type D personalities (marked by a generally sad and gloomy view of life and social inhibition) (Bartels et al., 2010a,b). The influence of this personality type on the severity of distress was partly mediated by anxiety and depression (also possibly reactions to tinnitus). Cognitive variables such as dysfunctional thoughts (Lee et al., 2004), particularly catastrophization could also influence this relationship (Weise et al., 2013), as could tinnitus-specific perceptions of illness (Vollmann et al., 2013). Dysfunctional coping strategies are related to an increase in tinnitus distress (Budd and Pugh, 1996; Scott and Lindberg, 2000). An increase in suspicions about external control reinforces the psychiatric symptoms associated with tinnitus (Budd et al., 1998).

The presence of a formal psychological disorder is another source of stress for the person. While psychiatric disturbance may be a reaction to tinnitus, it has also been proposed as a trigger for tinnitus (Henry et al., 2005). In 64% of cases, the onset of tinnitus is preceded by other mental disorders (Goebel and Floezinger, 2008). Studies point to a link between problematic tinnitus and depression (Wilson et al., 1991; Goebel and Floezinger, 2008), connecting the severity of tinnitus to that of depression or anxiety (Budd and Pugh, 1996; Andersson et al., 2005; Zoger et al., 2006). Accordingly, an improvement in depressive symptoms has been associated with a reduction of tinnitus distress (Folmer, 2002).

The literature offers the overall impression of a diathesis-stress interaction in which the diathesis is a patient's vulnerability to distress, and the stress is the level of tinnitus, an idea supported by Andersson and McKenna (1998); Kroner-Herwig et al. (2006). Patients who find tinnitus highly distressing often exhibit additional somatic conditions or comorbidities including autoimmune diseases, cardiovascular, endocrine, or metabolic diseases (Stobik et al., 2003). Increased tension of the head and neck muscles and other functional impairments have been described in tinnitus patients (Rubinstein et al., 1990; Peroz, 2003). These factors have raised clinical interest in a classification of tinnitus distress that takes into account not only the degree of tinnitus, hearing loss, and the audiological characteristics of the conditions but also psychosocial variables and comorbid factors (i.e., a biopsychosocial model) (Seydel et al., 2010).

Prior studies have linked single mental and physical factors to tinnitus distress; in the present study we carry out *simultaneous analyses* of its association with many factors, hoping to develop a broader view. The literature shows that stress, anxiety or depression contribute to tinnitus-related distress; here, we wanted to know if this distress is affected by *individual but networked conditions* that can be assessed by the subscales of psychometric instruments. Using a large data set, we also aimed to determine *where* such conditions overlap with tinnitus, as well as ways in which tinnitus-induced distress *differs* from the experience of other types of stress and emotions. Finally, we used quantitative methods to assign weights to the factors in terms of their relative contributions to tinnitus impairment.

## Methods

### Patients

Five hundred and thirty one patients with chronic tinnitus were recruited to this study, which was carried out between January 2008 and March 2010. The patients originated from a routine flow of individuals consecutively admitted to day ward of Tinnitus Center for treatment. The study sample consisted of 251 (47%) men and 280 (53%) women with a mean age of 49 years (SD 13.29 + Min 16 Max 59). All patients were informed of the purpose for which the data was being collected and gave their consent. This study was approved by the local Ethics Committee.

### Audiometry

Pure tone audiometry was performed on both ears of each patient to determine the degree and nature of any hearing loss. A discomfort threshold was used to determine the possible presence of hyperacusis; (data not included in the evaluation). We used tinnitus matching (frequency and loudness) to detect and provide an audiometric description of each patient's tinnitus.

### Psychometric evaluation

The study employed the self-reporting psychometric instruments shown in Table 1, chosen on the basis of clinical experience and representative cross-reference data from the Department of Psychosomatic Medicine, Charité, Universitätsmedizin Berlin (scores obtained from tinnitus patients are compared with those of patients with psychosomatic disorders in Zirke et al., 2013 and Devine et al., 2016).

- The degree of tinnitus distress was measured using the German version of tinnitus questionnaire TQ (Goebel and Hiller, 1994). The subscales include emotional and cognitive stress, intrusiveness of tinnitus, hearing problems, sleep disorders, and somatic symptoms associated with tinnitus.
- The Perceived Stress Questionnaire (Fliege et al., 2005) registers a subject's level and perception of stress (tension, worry, joy).
- Depressive symptoms were measured using the validated German version of the Center for Epidemiologic Studies Depression Scale, abbreviated here as ADS—Allgemeine Depression Skala (Radloff, 1991).
- Additional measurements were made of “anxious depression,” “annoyance,” and “positive mood” with the Berlin mood questionnaire (BSF) (Hoerhold and Klapp, 1993).
- Subjects' quality of life and mental and physical functions were assessed with the Short Form Health Survey (SF-36) (Morfeld et al., 2005).
- The Somatic Symptoms Inventory was used to characterize somatic symptoms considered independent of tinnitus.

**Table 1:** **Psychometric tests used during the study**.

**Questionnaire**	**Item example**	**Measured domain**
Tinnitus questionnaire by Goebel and Hiller (TQ, 51 items)	**Emotional stress:** to be worried sick	Tinnitus distress
	**Cognitive stress:** thoughts on prognosis	
	**Intrusiveness:** distraction of tinnitus	
	**Hearing problems:** distortion of voices	
	**Sleep disorders:** falling asleep	
	**Somatic symptoms:** severe headache	
Questionnaire to tinnitus localization and quality (TLQ, 10 Items)	**Localization:** left, **quality**: rustle	Tinnitus
Visual analog scales (VAS, 3 items)	**Loudness of tinnitus**	Tinnitus
	**Frequency of perception**: Affected by tinnitus	
Perceived stress questionnaire (PSQ; 20 items)	**General requirements:** They feel under deadline pressure	Stress amount
	**Tension:** You feel tense	Stress perception
	**Worries:** Your problems seem to pile up in front of you	
	**Joy:** You are a light hearted	
General depression scale (ADS-L; 20 Items)	**Depressive affect:** self-devaluation	Depression
	**Somatic symptoms**: drive	
	**Interpersonal experiences**: Rejection by others	
	**Positive affect:** zest for life	
Berlin mood questionnaire (BSF; 30 Items)	**Anxious depression:** I feel worried	Mood
	**Anger:** I feel aggressive	
	**Elation**: I feel solved	
	**Engagement:** I feel included	
Somatic symptoms inventory (BI; 24 Items)	**Fatigue:** fatigue	Somatic symptoms
	**Stomach complaints:** bloating	
	**Limb pain:** neck pain	
	**Heart disease:** shortness of breath	
Short form—8 health survey (SF8; 8 Items)	How much have you physical health or emotional problems in the past 4 weeks, limiting your normal contacts with family members or friends?	Quality of life
Total: 175 items		

Computational support included the use of a personal digital assistant (PDA) and data analysis, permitting physicians to provide subjects with an immediate interpretation of the results of their survey (Rose et al., 1999).

### Statistical evaluation

The analysis was performed with SPSS Statistics (version 15). First, the data were examined to determine the relationship between psychometric and audiological data and tinnitus distress (total tinnitus distress-score from TQ and its corresponding subscales). Correlations were calculated to provide initial values for the strength of each connection, followed by multiple regressions to examine cause and effect relationships. The dependent variable was TQ score whereas independent variables were the scores of remaining instruments and audiometric values. In addition to the psychometric tests, social data (age, sex, marital status, education, employment), and audiometric parameters such as hearing loss, tinnitus loudness, and frequency were included. This produced multiple regression model, which achieved a high level of significance, with an overall regression coefficient *R* = 0.993 (ANOVA: *F* = 24.753, *p* < 0.000). Due to limited capacity of SPSS version 15, the predictor importance was computed with a stochastically defined, randomly chosen by software subset of the sample (*N* = 140). In addition, multiple regression model was performed separately for the subset of 140 patients chosen for predictor importance computing. That model has also achieved high level of significance with regression coefficient *R* = 0.890 (ANOVA: *F* = 4.896, *p* < 0.000). All correlations that were significant for the whole sample remained significant in the subset and no new correlations were identified, indicative of sample homogeneity.

## Results

Based on the tinnitus distress score TQ, 400 patients (75%) were classified as having a compensated, moderate tinnitus response (TQ-score < 46), while 131 patients (25%) exhibited decompensated, severe tinnitus reactions (TQ score>, 47). The first interview recorded stress, depressive symptoms, disease-related fears, patients' medication and drug use, alongside audiometric measurements, and a medical history.

### Dependence of the degree of tinnitus distress (TQ) on somatic factors

#### Hearing

The audiometric examination revealed a moderate hearing loss of 23.53 dB/HL for the left ear and 22.96 dB/HL for the right ear in the study population.

#### Hearing loss

The correlation between hearing loss and tinnitus distress (TQ-score) was significant both for the total value of the TQ and the subscales “hearing problems” and “intrusiveness of tinnitus” (**Table 3**).

Multiple regressions (linear model, all variables) found no significant association between hearing loss, specific characteristics of tinnitus and the level of distress it caused. A tendency for a significant negative regression was found between hearing loss on the right and TQ-total value (*t* = −1.793, *p* = 0.075).

#### Tinnitus loudness

The loudness of tinnitus (dB HL, separated for unilateral and bilateral tinnitus), but not its frequency (Table 2), correlated with the scores of the TQ (Spearman correlation). Significant correlations were found between tinnitus loudness and the subscales “hearing problems” and “intrusiveness” and, with lower significance, for the total value of the TQ (Table 3). In the regression model, neither the loudness nor frequency of the tinnitus (respectively right/left) achieved significance.

**Table 2:** **Patients' description**.

	**Number of patients**	**Mean value**	**Standard deviation**	**Minimum**	**Maximum**
Age	531	48.88	13.29	16	79
Mean total score TQ	531	34.73	16.38	2	79
TQ emotional	531	9.53	5.49	0	23
TQ cognitive	531	6.13	3.90	0	16
TQ intrusiveness	531	9.51	3.55	0	16
TQ acoustic	531	4.70	3.55	0	14
TQ sleep	531	3.07	2.57	0	8
TQ somatic symptoms	531	1.79	1.74	0	6
PSQ worries	531	36.13	22.20	0	100
PSQ tension	531	50.83	22.88	0	100
PSQ joy	531	52.24	22.61	0	100
PSQ demands	531	44.91	24.17	0	100
Mean score ADSL	531	16.11	11.03	0	53
Tinnitus frequency right (Hz)	279	5058.17	2758.00	125	10,000
Tinnitus frequency left (Hz)	292	5380.14	2776.07	125	10,000
Tinnitus loudness right (dB)	276	36.09	21.17	−4	100
Tinnitus loudness left (dB)	290	37.42	20.97	2	93
Mean hearing loss right (dB)	531	23.00	16.73	1.9	130.0
Mean hearing loss left (dB)	530	23.28	15.01	0.6	86.4

**Table 3:** **Results of correlation analysis in the study population (***n*** = 531)**.

		**Tinnitus distress total score**	**Emotional and cognitive stress**	**Hearing problems**	**Somatic symptoms**	**Intrusiveness**	**Sleep problems**
**1. HEARING**							
Hearing loss right	*r_s_*	0.173^*^	0.090	0.247^*^	0.088	0.197^*^	−0.006
Hearing loss left	*r_s_*	0.235^**^	0.135^*^	0.384^**^	0.095	0.269^*^	0.064
**2. TINNITUS**							
Tinnitus loudness right	*r_s_*	0.176^*^	0.092	0.247^*^	0.083	0.227^**^	0.094
Tinnitus loudness left	*r_s_*	0.111^*^	0.027	0.239^**^	0.041	0.158^*^	0.039
Tinnitus frequency right	*r_s_*	0.008	−0.003	0.010	0.351^**^	0.426^**^	0.385^**^
Tinnitus frequency left	*r_s_*	−0.029	−0.018	−0.054	−0.065	−0.022	0.022
**3. SOMATIC SYMPTOMS INVENTORY BI**							
Fatigue	*r_s_*	0.535^**^	0.498^**^	0.359^**^	0.397^**^	0.424^**^	0.325^**^
Stomach	*r_s_*	0.403^**^	0.368^**^	0.359^**^	0.313^**^	0.283^**^	0.239^**^
Limbs	*r_s_*	0.478^**^	0.384^**^	0.403^**^	0.538^**^	0.373^**^	0.273^**^
Heart	*r_s_*	0.401^**^	0.358^**^	0.320^**^	0.330^**^	0.303^**^	0.225^**^
BI_total score	*r_s_*	0.588^**^	0.520^**^	0.449^*^	0.513^**^	0.452^**^	0.345^**^
**4. PSQ**							
Worries	*r_s_*	0.444^**^	0.472^**^	0.254^**^	0.226^*^	0.342^**^	0.208^**^
Joy	*r_s_*	−0.388^**^	−0.376^**^	−0.296^**^	−0.269^**^	−0.306^**^	−0.151^*^
Tension	*r_s_*	0.485^**^	0.442^**^	0.320^**^	0.328^**^	0.449^**^	0.270^**^
General requirements	*r_s_*	0.216^**^	0.196^*^	0.189^*^	0.162^*^	0.173^*^	0.083
PSQ_ total score	*r_s_*	0.463^**^	0.449^**^	0.322^**^	0.298^**^	0.382^**^	0.214^**^
**5. GENERAL DEPRESSION SCALE AND BERLIN MOOD QUESTIONNAIRE BSF, ADS**							
Engagement	*r_s_*	0.335^**^	−0.306^**^	−0.258^**^	0.239^**^	−0.269^**^	−0.178^*^
Joy	*r_s_*	−0.409^**^	−0.396^**^	−0.249^**^	−0.272^**^	−0.347^**^	−0.217^**^
Annoyance	*r_s_*	0.512^**^	0.518^**^	0.306^**^	0.311^**^	0.396^**^	0.275^**^
Anxious depression.	*r_s_*	0.586^**^	0.637^**^	0.283^**^	0.344^**^	0.426^**^	0.302^**^
ADS_ total score	*r_s_*	0.579^**^	0.572^**^	0.349^**^	0.351^**^	0.426^**^	0.385^**^
**6. SHORT FORM – 8 HEALTH SURVEY SF 8**							
Physical health	*r_s_*	−0.446^**^	0.417^**^	0.323^**^	−0.270^**^	−0.363^**^	−0.258^**^
Mental health	*r_s_*	−0.455^**^	−0.464^**^	0.295^**^	−0.249^**^	−0.339^**^	−0.240^**^
SF8_ total score	*r_s_*	−0.388^**^	−0.370^**^	−0.315^**^	−0.201^*^	−0.299^**^	−0.199^*^

*r_s_, correlation coefficient*.

* *, significance p < 0.05*.

** *, significance p < 0.01*.

#### Somatic symptoms inventory (*BI*)

A significant correlation was found between somatic symptoms measured by Somatic Symptoms Inventory, all subscales of TQ, and the total value (Table 3).

The regression analysis revealed a significant regression of fatigue with tinnitus distress (TQ total) with*t* = 2.038 (*p* = 0.043).

### Dependence of tinnitus distress (from TQ) on psychological burden

#### Stress (PSQ) related to tinnitus distress (TQ)

The level of perceived stress was significantly associated with tinnitus distress (TQ-scores) and correlated with the following PSQ subscales: demands, worry, tension, and joy (Table 3).

The regression analysis revealed no significant correlation in the overall model.

#### Depression score (ADS) correlates with tinnitus distress (TQ)

The total score for the Depression-Scale, which mainly focuses on cognitive aspects of depression such as a tendency to worry and negative thought circuits, correlated to the total TQ score as well as with its all separate subscales at a high level of significance (Table 3).

The multiple regression model revealed a significant regression with *t* = 2.98 (*p* = 0.003).

#### Mood scores (BSF) are related to tinnitus distress (TQ)

Symptoms of anxiety and depression, which were evaluated with the BSF, correlated significantly with the total TQ score and all its separate subscales, as was the case for “annoyance.” The positive moods, commitment and joy, had significant negative correlations with the total value and all subscales (Table 3).

Multiple regression found a significant relationship for anxious depression and total TQ with *t* = −2.20 (*p* = 0.029).

### Dependence of tinnitus distress (TQ) on social stress

#### Quality of life (SF-8) is significantly affected by tinnitus distress (TQ)

A significant negative correlation was found between physical and mental health as measured by the SF-8 and the total TQ score as well as all TQ subscales (Table 3). In the regression model, no significant correlations were found.

#### Age, gender, and marital status are related to tinnitus distress (TQ)

Patients who lived alone had a higher hearing loss on both sides and greater hearing problems than those living with a partner, as indicated by scores of the respective subscales (TQ). A similar trend was found for tinnitus loudness, judged higher by people living alone. The regression analysis showed a significant positive correlation with age *t* = 2.23 (*p* = 0.027).

### Weighting of each component in relation to tinnitus distress (TQ) in the regression analysis

**Figure 1:**
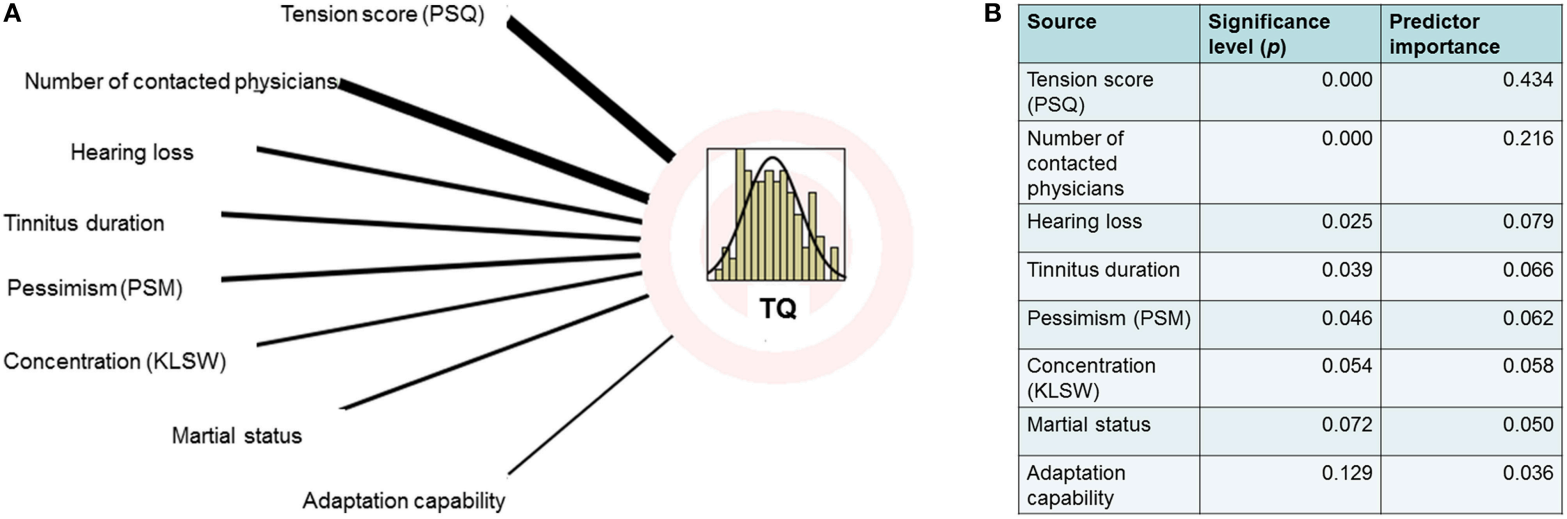
**(A)** Regression analysis graphical model [overall model: *R* = 0.993 (ANOVA: *F* = 322.97, *p* < 0.000)] calculated with subset of the data (*n* = 140). **(B)** Computed predictor importance and significance.

**Figure 2:**
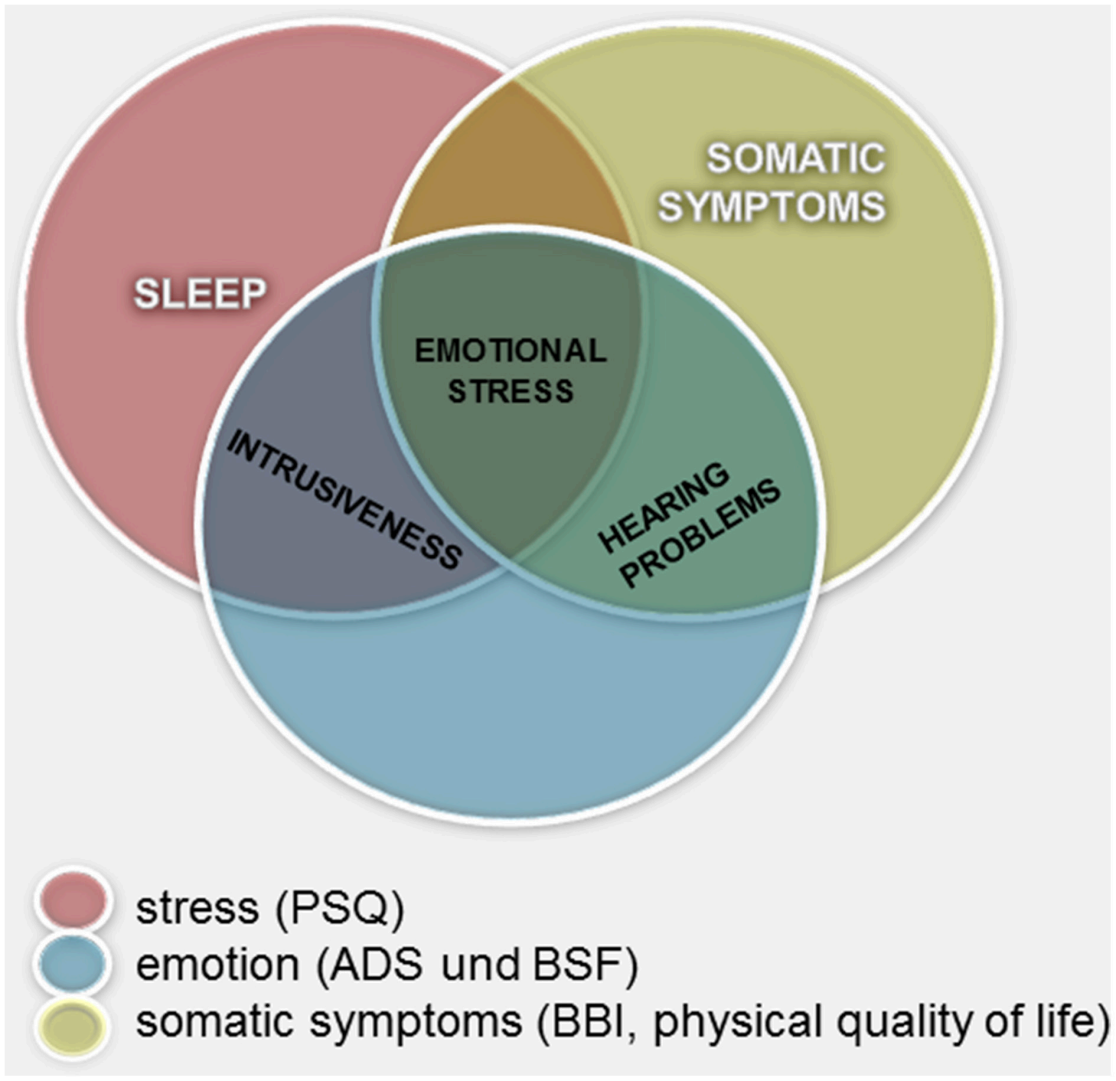
**Measurements of constructs related to tinnitus distress: graphical summary of the overlapping and not covering areas**.

The multiple regression analysis [evaluating the effects of tinnitus distress (TQ) for other psychometric data] showed that depression and stress scores had particularly high predictor values (Figures 1, 2).

The analysis of social variables revealed significant correlations with age, education, employment, and the number of physicians a patient had consulted. Additionally, a correlation, between hearing loss and tinnitus distress was found.

#### Summary of results

- There was a clear association between physical symptoms and tinnitus distress TQ, particularly seen in correlations with the TQ subscales “hearing problems” and “somatic symptoms,” and to some degree with “emotional stress.”
- Quality of life and tinnitus distress are negatively correlated, as indicated by the negative influence of the subscales “emotional stress,” “somatic symptoms,” and “hearing problems” on mental health.
- Tinnitus distress (TQ total score) correlated significantly with perceived stress and (depressive) mood. For perceived stress (PSQ), the TQ subscales “emotional stress” and “hearing problems” proved to be especially significant. The subscales “emotional stress,” “sleep problems” and, to a lesser degree, “tinnitus intrusiveness” significantly correlated with the depression grade.

## Discussion

The aim of our study was to simultaneously measure associations between tinnitus distress and other mental and physical conditions in a single group of patients. Previous studies have linked distress to a number of individual factors, but their relative contributions to the outcome of tinnitus distress have not been ranked in a single model. Our efforts found a number of correlations between measurements of tinnitus distress (TQ) and physical symptoms, the quality of life, perceived stress, and depressive symptoms.

Psychometric methods have been established as a means of expressing tinnitus distress. A number of studies have concluded that psychoacoustic features of tinnitus, such as loudness, cannot be used to predict a patient's degree of distress (Henry and Meikle, 2000; Hiller and Goebel, 2007). Here we demonstrate that acoustic variables such as the loudness and frequency of tinnitus correlate only marginally with the overall tinnitus-related burden experienced by patients. Tinnitus loudness could only be correlated with the TQ subscales “hearing problems” and “intrusiveness of tinnitus.” The louder the tinnitus, the more penetrating is its perception. However, our study revealed no correlation was found between tinnitus frequency and the TQ total score.

Past studies have reported some correlations between auditory processing and patient mood. A representative study recently conducted in Sweden concluded that the strongest predictor for the *occurrence* of tinnitus was hearing loss. A patient's subjective *experience* of tinnitus, however, was more dependent on the presence of a depressive co-morbidity (Hebert et al., 2012; Kraus and Canlon, 2012).

In earlier work (Mazurek et al., 2010b), we determined that tinnitus-induced distress is positively correlated with hearing loss, and the present study corroborates this finding.

We also found that age has a significant impact on tinnitus distress. This confirms findings from single-factor association studies that have linked distress to hearing problems and sleep disorders, which also increase with age. Similar findings from other recent studies demonstrate that older patients find tinnitus more stressful (Schlee et al., 2011a). Previous work by our group has shown that age influences various aspects of tinnitus distress; starting at the age of 60, patients report more distress (Seydel et al., 2013).

Here our computing of predictor importance identifies only two factors that account for a significant amount of the variance of tinnitus distress: the side on which patients experience tinnitus, and the overall status of their hearing. No significant correlations were found based on age or gender.

We have shown that tinnitus distress is closely related to more general types of stress, a negative mood, and hearing loss. Increasing age is generally accompanied by poorer hearing and other somatic symptoms, which then amplify tinnitus distress. Age-related changes in tinnitus have been reported in the literature (see a review Henry et al., 2005) and in detail by our group (Seydel et al., 2013, 2015). The correlations that emerge, however, are multidimensional. Causal relationships seem to be indicated for gender, the duration of tinnitus, hearing impairment, age, and psychosocial variables (such as employment, education level, and use of the health system).

An analysis of the influence of marital status of tinnitus patients revealed a trend toward greater hearing loss in people who live alone. They perceive tinnitus louder than patients who live with a partner. Somatic symptoms and general physical health (quality of life-SF 8) are co-morbidities whose effects vary significantly in association with hearing, and partly in relation to age and sex. A decline in hearing had a significant influence on other symptoms captured in the somatic symptoms inventory (BI). As people age, they generally report a decline in aspects of the quality of life linked to physical health. For tinnitus patients, the loss of hearing likely has an additive effect on these negative perceptions.

A review of associations that have been made between tinnitus and subjective tinnitus distress in the literature yielded high correlations with “somatic problems” and “intrusiveness.” This association seems to depend on a patient's perception of tinnitus and its additive effects on other somatic problems, some of which may have a physiological basis. Liotti and Mayberg have proposed that a negative mood or depressive emotions, which chiefly originate in the limbic system, can lead to down-regulations of activity in the inferior parietal cortex and dorsolateral prefrontal cortex (Liotti and Mayberg, 2001). These regions include the auditory cortex, where acoustic stimuli are processed. The effects may extend to a wider network involved in attention and spatial orientation (Sturm et al., 2004; Schonwiesner et al., 2007; McKenna et al., 2014). By modulating processes and brain areas involved in attention, negative moods may also influence activity in the auditory or somatosensory areas of the brain.

Previous studies have reported high correlations between the total score for tinnitus distress (as determined by the -TQ) with stress and stress perception (Olderog et al., 2004), with depression and negative mood (Andersson and Kaldo, 2004), and even with physical complaints and perceptions of a decline in quality of life (Cima et al., 2011). Bi-directional interference has been observed between tinnitus perception and stress, pain, sleep, and fatigue Hebert et al. (2012). Langguth (2011) suggest that there are similarities in the pathophysiology of depression and tinnitus, especially in regard to associated distress factors (Langguth, 2011). Here we confirm a strong correlation between tinnitus distress and other forms of stress and depression. However, the regression analysis offers a multi-dimensional view of these relationships, suggesting that other forms of somatic stress and depression contribute to total tinnitus distress (TQ) in distinct ways. The stress scales (PSQ) showed correlations to all subscales of TQ; correlation coefficients were particularly high for emotional stress, intrusiveness and tinnitus-induced hearing problems. Depression, on the other hand, was predominantly correlated with the TQ-subscales “emotional stress,” “intrusiveness,” and “sleep.”

So far these studies have exposed a number of factors that play some role in the development of tinnitus and the ways patients cope, but they have not turned up a specific association that correlates with a high-distress response reliably enough to serve as a predictor. This will come as no surprise to clinicians, who are familiar with the diverse symptoms and responses of patients. What has been missing is a more complex model that integrates these findings, reveals combinations of factors that influence the facets of tinnitus, and assigns weights to components that reflect their contributions to various processes.

Our approach involved a large cohort of tinnitus patients, from whom we simultaneously captured a wide range of psychological and somatic parameters. We used computational methods to detect associations between high tinnitus distress and various combinations of psychological, biological, and lifestyle factors. The analysis revealed a network of associations linking aspects of tinnitus distress to other aspects of patients' lives and health. In some cases we could assess the contributions of each component and distinguish crucial factors from weak contributors.

We anticipated that our combined approach would reveal new, more precise associations between subscales of tinnitus distress and diverse aspects of patients' lives; these, in turn, could provide new insights into causal mechanisms that linked them. Finding a strong correlation between tinnitus perception and depression and negative moods, for example, is evidence of a connection between acoustic perception and the regulation of emotions, thus implicating specific regions of the brain and possibly suggesting something about their coordination.

The Global Brain Model of Tinnitus proposes a scenario in which acoustic input to the brain falls, which can be caused by various types of damage to the auditory system (Schlee et al., 2011b). This leads to a change in the central auditory system, where inhibitory mechanisms usually dampen the strength of acoustic signals; now those mechanisms become less active, resulting in more excitation of cortical areas.

The activity of auditory areas is modulated by a fronto-parietal-cingular network, where higher activity is associated with high levels of patient distress. The particularly important structures in this network are the dorsolateral prefrontal cortex, the orbitofrontal cortex, the anterior cingulum, and the precuneus/posterior cingulate. De Ridder et al. (2011) describes a network of ACC, amygdala, and anterior insula (tested by EEG data) and claims that it is responsible for tinnitus distress. This network is also activated in pain-associated distress (Moisset and Bouhassira, 2007). Recent epidemiological studies suggest that similar or related symptoms might account for the relationship between tinnitus distress and depression, but they may also have common etiologies (Hebert et al., 2012). These ideas are interesting in light of the observation that either depression or a high general level of stress leads to a more pronounced perception of tinnitus symptoms by patients (Bartels et al., 2010b).

The diverse models that have been proposed for tinnitus distress may indicate that it might be reached through several routes that depend on different mechanisms. Clinical experience would tend to support this view, given the diversity of patients affected by tinnitus and their subjective experiences of it. Ultimately, hypotheses and models of tinnitus mechanisms must be validated in patient studies, and ideally they should be designed with an eye to clinical benefits and new, effective types of treatment.

Further studies will be necessary to clarify how different processing mechanisms interact to produce the complex symptoms of tinnitus. At least three types of components are involved: somatic (generators, influenced by a range of somatosensory stimuli), psychological (emotional “depressive” processing, attention, stress), and social. The unique features of tinnitus might trigger a unique “Distress Network”; this might explain the increased level of stress and other physical and psychological symptoms observed in patients with chronic decompensated tinnitus (De Ridder et al., 2011). Helping patients become habituated to tinnitus and find healthy ways to compensate will probably require therapies that address and reduce combinations of symptoms related to stress and depression.

## Author contributions

PB: study conception and design; data acuisition; analysis and interpretation of data; drafting of manuscript. AS: analysis and interpretation of data; drafting of manuscript. MR: analysis and interpretation of data. LM: critical revision; drafting of manuscript. HO: critical revision. BM: study design; data acuisition; analysis and interpretation of data.

### Conflict of interest statement

The authors declare that the research was conducted in the absence of any commercial or financial relationships that could be construed as a potential conflict of interest.

## References

[B1] Andersson G. Airikka M. Buhrman M. Kaldo V. (2005). Dimensions of perfectionism and tinnitus distress. Psychol. Health Med. 10, 78–87. 10.1080/13548500512331315389

[B2] Andersson G. Kaldo V. (2004). Internet-based cognitive behavioral therapy for tinnitus. J. Clin. Psychol. 60, 171–178. 10.1002/jclp.10243 14724924

[B3] Andersson G. McKenna L. (1998). Tinnitus masking and depression. Audiology 37, 174–182. 10.3109/00206099809072971 9626862

[B4] Andersson G. Westin V. (2008). Understanding tinnitus distress: introducing the concepts of moderators and mediators. Int. J. Audiol. 47(Suppl. 2), S106–S111. 10.1080/14992020802301670 19012118

[B5] Bartels H. Middel B. Pedersen S. S. Staal M. J. Albers F. W. (2010a). The distressed (Type D) personality is independently associated with tinnitus: a case-control study. Psychosomatics 51, 29–38. 10.1176/appi.psy.51.1.29 20118438

[B6] Bartels H. Pedersen S. S. Van Der Laan B. F. Staal M. J. Albers F. W. Middel B. (2010b). The impact of Type D personality on health-related quality of life in tinnitus patients is mainly mediated by anxiety and depression. Otol. Neurotol. 31, 11–18. 10.1097/MAO.0b013e3181bc3dd1 19816233

[B7] Biesinger E. Reisshauer A. Mazurek B. (2008). [The role of the cervical spine and the craniomandibular system in the pathogenesis of tinnitus. Somatosensory tinnitus]. HNO 56, 673–677. 10.1007/s00106-008-1721-2 18560742

[B8] Budd R. J. Oles G. Hughes I. C. (1998). The relationship between coping style and burden in the carers of relatives with schizophrenia. Acta Psychiatr. Scand. 98, 304–309. 10.1111/j.1600-0447.1998.tb10088.x 9821452

[B9] Budd R. J. Pugh R. (1996). Tinnitus coping style and its relationship to tinnitus severity and emotional distress. J. Psychosom. Res. 41, 327–335. 10.1016/S0022-3999(96)00171-7 8971662

[B10] Chrousos G. P. (2009). Stress and disorders of the stress system. Nat. Rev. Endocrinol. 5, 374–381. 10.1038/nrendo.2009.106 19488073

[B11] Cima R. F. Crombez G. Vlaeyen J. W. (2011). Catastrophizing and fear of tinnitus predict quality of life in patients with chronic tinnitus. Ear Hear. 32, 634–641. 10.1097/AUD.0b013e31821106dd 21399500

[B12] De Ridder D. Elgoyhen A. B. Romo R. Langguth B. (2011). Phantom percepts: tinnitus and pain as persisting aversive memory networks. Proc. Natl. Acad. Sci. U.S.A. 108, 8075–8080. 10.1073/pnas.1018466108 21502503 PMC3100980

[B13] Devine J. Fliege H. Kocalevent R. Mierke A. Klapp B. F. Rose M. (2016). Evaluation of Computerized Adaptive Tests (CATs) for longitudinal monitoring of depression, anxiety, and stress reactions. J. Affect. Disord. 190, 846–853. 10.1016/j.jad.2014.10.063 25481813

[B14] Eggermont J. J. Roberts L. E. (2012). The neuroscience of tinnitus: understanding abnormal and normal auditory perception. Front. Syst. Neurosci. 6:53. 10.3389/fnsys.2012.00053 22798948 PMC3394370

[B15] Fliege H. Rose M. Arck P. Walter O. B. Kocalevent R. D. Weber C.. (2005). The Perceived Stress Questionnaire (PSQ) reconsidered: validation and reference values from different clinical and healthy adult samples. Psychosom. Med. 67, 78–88. 10.1097/01.psy.0000151491.80178.78 15673628

[B16] Folmer R. L. (2002). Long-term reductions in tinnitus severity. BMC Ear Nose Throat Disord. 2:3. 10.1186/1472-6815-2-3 12234379 PMC128822

[B17] Goebel G. Floezinger U. (2008). Pilot study to evaluate psychiatric co-morbidity in tinnitus patients with and without hyperacusis. Audiol. Med. 6, 78–84. 10.1080/16513860801959100

[B18] Goebel G. Hiller W. (1994). [The tinnitus questionnaire. A standard instrument for grading the degree of tinnitus. Results of a multicenter study with the tinnitus questionnaire]. HNO 42, 166–172. 8175381

[B19] Halford J. B. Anderson S. D. (1991). Anxiety and depression in tinnitus sufferers. J. Psychosom. Res. 35, 383–390. 10.1016/0022-3999(91)90033-K 1920169

[B20] Hallam R. S. McKenna L. Shurlock L. (2004). Tinnitus impairs cognitive efficiency. Int. J. Audiol. 43, 218–226. 10.1080/14992020400050030 15250126

[B21] Hebert S. Canlon B. Hasson D. Magnusson Hanson L. L. Westerlund H. Theorell T. (2012). Tinnitus severity is reduced with reduction of depressive mood–a prospective population study in Sweden. PLoS ONE 7:e37733. 10.1371/journal.pone.0037733 22629449 PMC3358289

[B22] Henry J. A. Dennis K. C. Schechter M. A. (2005). General review of tinnitus: prevalence, mechanisms, effects, and management. J. Speech Lang. Hear. Res. 48, 1204–1235. 10.1044/1092-4388(2005/084) 16411806

[B23] Henry J. A. Meikle M. B. (2000). Psychoacoustic measures of tinnitus. J. Am. Acad. Audiol. 11, 138–155. 10755810

[B24] Henry J. L. Wilson P. H. (1995). Coping with tinnitus: two studies of psychological and audiological characteristics of patients with high and low tinnitus-related distress. Int. Tinnitus J. 1, 85–92. 10753328

[B25] Hesse G. (2008). [Neurootologic and psychosomatic habituation therapy. Treatment approaches in chronic tinnitus]. HNO 56, 686–693. 10.1007/s00106-008-1723-0 18560741

[B26] Hesser H. Andersson G. (2009). The role of anxiety sensitivity and behavioral avoidance in tinnitus disability. Int. J. Audiol. 48, 295–299. 10.1080/14992020802635325 19842804

[B27] Hiller W. Goebel G. (1992). A psychometric study of complaints in chronic tinnitus. J. Psychosom. Res. 36, 337–348. 10.1016/0022-3999(92)90070-I 1593509

[B28] Hiller W. Goebel G. (2007). When tinnitus loudness and annoyance are discrepant: audiological characteristics and psychological profile. Audiol. Neurootol. 12, 391–400. 10.1159/000106482 17664870

[B29] Hoerhold M. Klapp B. (1993). Testing the invariance and hierarchy of a multidimensional model of mood by means of repeated measurement with student and patient samples. Z. Med. Psychol. 2, 27–35.

[B30] Horner K. C. (2003). The emotional ear in stress. Neurosci. Biobehav. Rev. 27, 437–446. 10.1016/S0149-7634(03)00071-X 14505685

[B31] Jastreboff P. J. (1999). Tinnitus retraining therapy. Br. J. Audiol. 33, 68–70. 10219725

[B32] Kleinstauber M. Jasper K. Schweda I. Hiller W. Andersson G. Weise C. (2013). The role of fear-avoidance cognitions and behaviors in patients with chronic tinnitus. Cogn. Behav. Ther. 42, 84–99. 10.1080/16506073.2012.717301 23199238

[B33] Kraus K. S. Canlon B. (2012). Neuronal connectivity and interactions between the auditory and limbic systems. Effects of noise and tinnitus. Hear. Res. 288, 34–46. 10.1016/j.heares.2012.02.009 22440225

[B34] Kroner-Herwig B. Zachriat C. Weigand D. (2006). Do patient characteristics predict outcome in the outpatient treatment of chronic tinnitus? Psychosoc. Med. 3:Doc07. 19742075 PMC2736505

[B35] Langenbach M. Olderog M. Michel O. Albus C. Kohle K. (2005). Psychosocial and personality predictors of tinnitus-related distress. Gen. Hosp. Psychiatry 27, 73–77. 10.1016/j.genhosppsych.2004.08.008 15694221

[B36] Langguth B. (2011). A review of tinnitus symptoms beyond ‘ringing in the ears’: a call to action. Curr. Med. Res. Opin. 27, 1635–1643. 10.1185/03007995.2011.595781 21699365

[B37] Leaver A. M. Renier L. Chevillet M. A. Morgan S. Kim H. J. Rauschecker J. P. (2011). Dysregulation of limbic and auditory networks in tinnitus. Neuron 69, 33–43. 10.1016/j.neuron.2010.12.002 21220097 PMC3092532

[B38] Lee S. Y. Kim J. H. Hong S. H. Lee D. S. (2004). Roles of cognitive characteristics in tinnitus patients. J. Korean Med. Sci. 19, 864–869. 10.3346/jkms.2004.19.6.864 15608399 PMC2816302

[B39] Liotti M. Mayberg H. S. (2001). The role of functional neuroimaging in the neuropsychology of depression. J. Clin. Exp. Neuropsychol. 23, 121–136. 10.1076/jcen.23.1.121.1223 11320448

[B40] Ma Y. L. Gerhardt K. J. Curtis L. M. Rybak L. P. Whitworth C. Rarey K. E. (1995). Combined effects of adrenalectomy and noise exposure on compound action potentials, endocochlear potentials and endolymphatic potassium concentrations. Hear. Res. 91, 79–86. 10.1016/0378-5955(95)00172-7 8647728

[B41] Mazurek B. Haupt H. Joachim R. Klapp B. F. Stover T. Szczepek A. J. (2010a). Stress induces transient auditory hypersensitivity in rats. Hear. Res. 259, 55–63. 10.1016/j.heares.2009.10.006 19840840

[B42] Mazurek B. Haupt H. Klapp B. F. Szczepek A. J. Olze H. (2012). Exposure of Wistar rats to 24-h psycho-social stress alters gene expression in the inferior colliculus. Neurosci. Lett. 527, 40–45. 10.1016/j.neulet.2012.08.019 22922217

[B43] Mazurek B. Olze H. Haupt H. Szczepek A. J. (2010b). The more the worse: the grade of noise-induced hearing loss associates with the severity of tinnitus. Int. J. Environ. Res. Public Health 7, 3071–3079. 10.3390/ijerph7083071 20948948 PMC2954569

[B44] McKenna L. Handscomb L. Hoare D. J. Hall D. A. (2014). A scientific cognitive-behavioral model of tinnitus: novel conceptualizations of tinnitus distress. Front. Neurol. 5:196. 10.3389/fneur.2014.00196 25339938 PMC4186305

[B45] Milerova J. Anders M. Dvorak T. Sand P. G. Koniger S. Langguth B. (2013). The influence of psychological factors on tinnitus severity. Gen. Hosp. Psychiatry 35, 412–416. 10.1016/j.genhosppsych.2013.02.008 23602606

[B46] Moisset X. Bouhassira D. (2007). Brain imaging of neuropathic pain. Neuroimage 37(Suppl. 1), S80–S88. 10.1016/j.neuroimage.2007.03.054 17512757

[B47] Morfeld M. Bullinger M. Nantke J. Brahler E. (2005). [The version 2.0 of the SF-36 health survey: results of a population-representative study]. Soz. Praventivmed. 50, 292–300. 10.1007/s00038-005-4090-6 16300173

[B48] Nondahl D. M. Cruickshanks K. J. Huang G. H. Klein B. E. Klein R. Tweed T. S.. (2012). Generational differences in the reporting of tinnitus. Ear Hear. 33, 640–644. 10.1097/AUD.0b013e31825069e8 22588269 PMC3422442

[B49] Olderog M. Langenbach M. Michel O. Brusis T. Kohle K. (2004). [Predictors and mechanisms of tinnitus distress - a longitudinal analysis]. Laryngorhinootologie 83, 5–13. 10.1055/s-2004-814235 14740299

[B50] Pattyn T. Van Den Eede F. Vanneste S. Cassiers L. Veltman D. J. Van De Heyning P.. (2016). Tinnitus and anxiety disorders: a review. Hear. Res. 333, 255–265. 10.1016/j.heares.2015.08.014 26342399

[B51] Peroz I. (2003). [Dysfunctions of the stomatognathic system in tinnitus patients compared to controls]. HNO 51, 544–549. 10.1007/s00106-002-0750-5 12904875

[B52] Radloff L. S. (1991). The use of the Center for Epidemiologic Studies Depression Scale in adolescents and young adults. J. Youth Adolesc. 20, 149–166. 10.1007/BF01537606 24265004

[B53] Rauschecker J. P. Leaver A. M. Muhlau M. (2010). Tuning out the noise: limbic-auditory interactions in tinnitus. Neuron 66, 819–826. 10.1016/j.neuron.2010.04.032 20620868 PMC2904345

[B54] Rose M. Hess V. Horhold M. Brahler E. Klapp B. F. (1999). [Mobile computer-assisted psychometric diagnosis. Economic advantages and results on test stability]. Psychother. Psychosom. Med. Psychol. 49, 202–207. 10416340

[B55] Rubinstein B. Axelsson A. Carlsson G. E. (1990). Prevalence of signs and symptoms of craniomandibular disorders in tinnitus patients. J. Craniomandib. Disord. 4, 186–192. 2098394

[B56] Schlee W. Kleinjung T. Hiller W. Goebel G. Kolassa I. T. Langguth B. (2011a). Does tinnitus distress depend on age of onset? PLoS ONE 6:e27379. 10.1371/journal.pone.0027379 22125612 PMC3220697

[B57] Schlee W. Lorenz I. Hartmann T. Müller N. Schulz H. Weisz N. (2011b). A global brain model of tinnitus, in Textbook of Tinnitus, eds Møller A. R. Langguth B. De Ridder D. Kleinjung T. (New York, NY: Springer New York), 161–169.

[B58] Schonwiesner M. Novitski N. Pakarinen S. Carlson S. Tervaniemi M. Naatanen R. (2007). Heschl's gyrus, posterior superior temporal gyrus, and mid-ventrolateral prefrontal cortex have different roles in the detection of acoustic changes. J. Neurophysiol. 97, 2075–2082. 10.1152/jn.01083.2006 17182905

[B59] Scott B. Lindberg P. (2000). Psychological profile and somatic complaints between help-seeking and non-help-seeking tinnitus subjects. Psychosomatics 41, 347–352. 10.1176/appi.psy.41.4.347 10906357

[B60] Seydel C. Haupt H. Olze H. Szczepek A. J. Mazurek B. (2013). Gender and chronic tinnitus: differences in tinnitus-related distress depend on age and duration of tinnitus. Ear Hear. 34, 661–672. 10.1097/AUD.0b013e31828149f2 23439056

[B61] Seydel C. Haupt H. Szczepek A. J. Hartmann A. Rose M. Mazurek B. (2015). Three years later: report on the state of well-being of patients with chronic tinnitus who underwent modified tinnitus retraining therapy. Audiol. Neurootol. 20, 26–38. 10.1159/000363728 25413891

[B62] Seydel C. Haupt H. Szczepek A. J. Klapp B. F. Mazurek B. (2010). Long-term improvement in tinnitus after modified tinnitus retraining therapy enhanced by a variety of psychological approaches. Audiol. Neurootol. 15, 69–80. 10.1159/000231632 19657182

[B63] Stobik C. Weber R. K. Munte T. F. Frommer J. (2003). [Psychosomatic stress factors in compensated and decompensated tinnitus]. Psychother. Psychosom. Med. Psychol. 53, 344–352. 10.1055/s-2003-40947 12886492

[B64] Sturm W. Longoni F. Fimm B. Dietrich T. Weis S. Kemna S.. (2004). Network for auditory intrinsic alertness: a PET study. Neuropsychologia 42, 563–568. 10.1016/j.neuropsychologia.2003.11.004 14725794

[B65] Vollmann M. Scharloo M. Langguth B. Kalkouskaya N. Salewski C. (2013). Illness representations as mediators of the relationship between dispositional optimism and depression in patients with chronic tinnitus: a cross-sectional study. Psychol. Health 29, 81–93. 10.1080/08870446.2013.828294 23937149

[B66] Wallhausser-Franke E. Brade J. Balkenhol T. D'amelio R. Seegmuller A. Delb W. (2012). Tinnitus: distinguishing between subjectively perceived loudness and tinnitus-related distress. PLoS ONE 7:e34583. 10.1371/annotation/96f457f9-3f48-4f88-a7f0-1d5e6067e7a5 22529921 PMC3329489

[B67] Weise C. Hesser H. Andersson G. Nyenhuis N. Zastrutzki S. Kroner-Herwig B.. (2013). The role of catastrophizing in recent onset tinnitus: its nature and association with tinnitus distress and medical utilization. Int. J. Audiol. 52, 177–188. 10.3109/14992027.2012.752111 23301660

[B68] Wilson P. H. Henry J. Bowen M. Haralambous G. (1991). Tinnitus reaction questionnaire: psychometric properties of a measure of distress associated with tinnitus. J. Speech Hear. Res. 34, 197–201. 10.1044/jshr.3401.197 2008074

[B69] Wilson P. H. Henry J. L. (1998). Tinnitus cognitions questionnaire: development and psychometric properties of a measure of dysfunctional cognitions associated with tinnitus. Int. Tinnitus J. 4, 23–30. 10753381

[B70] Zenner H. P. (2003). [Cognitive tinnitus desensitization: evidence-based and guideline-adherent habituation therapy for chronic tinnitus sensitization]. HNO 51, 687–689. 10.1007/s00106-003-0939-2 12955244

[B71] Zirke N. Seydel C. Szczepek A. J. Olze H. Haupt H. Mazurek B. (2013). Psychological comorbidity in patients with chronic tinnitus: analysis and comparison with chronic pain, asthma or atopic dermatitis patients. Qual. Life Res. 22, 263–272. 10.1007/s11136-012-0156-0 22430181

[B72] Zoger S. Svedlund J. Holgers K. M. (2006). Relationship between tinnitus severity and psychiatric disorders. Psychosomatics 47, 282–288. 10.1176/appi.psy.47.4.282 16844885

